# Advancing lung organoids toward clinical applications: a global perspective on research focus and future directions

**DOI:** 10.3389/fmed.2025.1611304

**Published:** 2025-07-16

**Authors:** Qian Wang, Shiyan Tan, Xi Fu, Jiawei He, Qiong Ma, Fengming You, Liting You, Yifeng Ren

**Affiliations:** ^1^Hospital of Chengdu University of Traditional Chinese Medicine, Chengdu, China; ^2^TCM Regulating Metabolic Diseases Key Laboratory of Sichuan Province, Hospital of Chengdu University of Traditional Chinese Medicine, Chengdu, China; ^3^Department of Laboratory Medicine, West China Hospital, Sichuan University, Chengdu, China

**Keywords:** lung organoids, clinical applications, research focus, future directions, bibliometric

## Abstract

**Background:**

Lung organoids have emerged as a promising tool for studying lung development, function, and disease pathology. The present study aimed to analyze the current status and development trends of lung organoid research over the past years, present visual representations, and provide references for future research directions using bibliometric analysis.

**Methods:**

Information on articles on lung organoids extracted from the Web of Science Core Collection, such as year of publication, journal, country, institution, author, and keywords, was analyzed. R, VOSviewer, and SCImago Graphica were used to visualize publication trends, co-authorship analysis, co-occurrence analysis, and hotspot evolution.

**Results:**

The number of global publications has increased from 1 in 2011 to 929 in 2024. The Nature produced the highest number of citations (2,675 citations). The United States (8,155 citations and 281 publications), University Medical Center Utrecht (2083 citations and 11 publications), and Clevers H (2,711 citations and 21 publications) were the most influential countries, institutions, and authors, respectively. Co-occurrence cluster analysis of the top 54 keywords formed four clusters: (1) idiopathic pulmonary fibrosis, (2) lung cancer, (3) cystic fibrosis, (4) COVID-19.

**Conclusion:**

Overall, research on lung organoids continues to increase. The United States of America and the Netherlands dominated global studies. The analysis of pulmonary fibrosis, lung cancer and COVID-19 occupied a prominent position of research in this area. The research hotspots for lung organoids are disease model and microphysiological systems. Standardization, accurate disease modeling, and ethics and safety remain pressing challenges that need to be addressed in this field.

## Graphical abstract

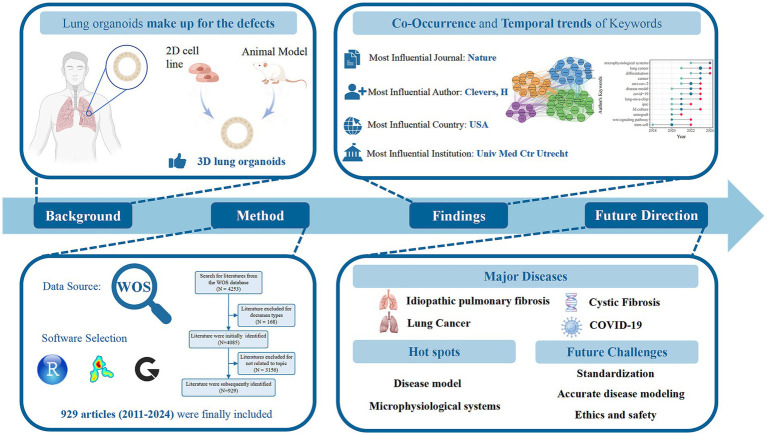

## 1 Introduction

The lung system includes airways and alveoli, facilitating immune defense and gas exchange. While traditional *in vitro* cell experiments and animal models have been used to study lung development and diseases, they have limitations. *In vitro* models fail to replicate human tissue structure and function, and animal models differ from human lungs, complicating clinical applications. Organoids are three-dimensional (3D) tissue-like structures created from adult or pluripotent stem cells *in vitro*. They closely mimic the architecture and functions of their source organs, making them the most precise organ models (1). These structures resemble the original organ, contain diverse cell types, and can self-renew to replicate some organ functions (2). It is more physiologically relevant than the monolayer culture model and easier to manipulate niche components, signaling pathways, and genome editing than animal models. In lung disease research, lung organoids address limitations of traditional models and connect basic research with clinical applications (3–5). They have gained prominence, especially during the corona virus disease 2019 (COVID-19) pandemic, as effective *in vitro* models for studying lung development, function, and disease.

To date, many review articles have focused on lung organoids, covering their creation, culture methods, and clinical uses (6–9). However, these often rely on subjective views and lack a comprehensive overview, leading to bias. Additionally, they fail to clearly outline research distribution in the field or provide timely, targeted guidance for clinical practice through interdisciplinary collaboration. The clinical use of lung organoids is hindered by challenges like standardizing culture methods and addressing ethical issues, which current research often overlooks. To navigate this complexity, a thorough evaluation of the growing scientific literature is needed, providing a clear understanding of the current research landscape and data-driven insights to guide future research priorities.

Bibliometrics is a scientific method that emphasizes comprehensive and adaptable analysis to map knowledge areas, highlight interdisciplinary work, and identify key advancements (10). Despite progress in lung organoid research, significant knowledge gaps remain. Bibliometric analysis can direct researchers to unexplored areas and encourage research to close these gaps. Besides, it can indirectly influence clinical research, promoting evidence-based practices for better disease management (11). Table 1 summarizes prior organoid studies employing bibliometric methods, highlighting differing topics despite similar methodologies. To our knowledge, this is the first visual analysis on lung organoids, and aimed to address the following questions:

**Table 1:** List of papers using bibliometrics as the research method.

References	Fields
Global Trends of Organoid and Organ-On-a-Chip in the Past Decade: A Bibliometric and Comparative Study (105)	Organoid and Organ-On-a-Chip
Current Trends and Research Topics Regarding Intestinal Organoids: An Overview Based on Bibliometrics (106)	Intestinal Organoids
Knowledge graphs of ethical concerns of cerebral organoids (107)	Ethical Concerns of Cerebral Organoids
Global Trends of Stem Cell Precision Medicine Research (2018–2022): A Bibliometric Analysis (108)	Stem Cell Precision Medicine
Patent bibliometric analysis for global trend of organoid technologies in the past decade (109)	Organoid Technologies
Global trends and hotspots in research on organoids between 2011 and 2020: a bibliometric analysis (110)	Organoids
Progress of research on tumor organoids: A bibliometric analysis of relevant publications from 2011 to 2021 (111)	Tumor organoids
Development trends of human organoid-based COVID-19 research based on bibliometric analysis (112)	Human organoid and COVID-19
Mapping the scientific output of organoids for animal and human modeling infectious diseases: a bibliometric assessment (113)	Organoids and infectious diseases
Human brain organoid: trends, evolution, and remaining challenges (114)	Brain organoid
Applications of 3D organoids in toxicological studies: a comprehensive analysis based on bibliometrics and advances in toxicological mechanisms (115)	Organoids and toxicity
Bibliometric and visualized analysis of hydrogels in organoids research (116)	Organoids and hydrogels
Interpretation of the past, present, and future of organoid technology: an updated bibliometric analysis from 2009 to 2024 (117)	Organoid technology

*Q1*. What are the global publishing trends and cooperation modes of lung organoids?

*Q2*. What is the main knowledge structure in the field of lung organoids?

*Q3*. What are the future prospectives and challenges in the field of lung organoid research?

We hope that this comprehensive bibliometric analysis can help researchers explore this promising research area.

## 2 Materials and methods

### 2.1 Literature search strategy

We selected the Web of Science Core Collection (WoSCC) for its high-quality, multidisciplinary coverage, making it ideal for bibliometric analysis. Our search strategy was set as follows: TS = (lung* OR pulmo*) AND TS = (((organoid*) OR (spheroid*)) OR (((3D) OR (three dimensions) OR (3 dimensions) OR (three dimensional) OR (3 dimensional)) AND (cell OR tissue) AND ((culture) OR (cultures) OR (cultured)))), focusing on English-language articles and reviews. The search covered the period from the database’s inception to December 31, 2024. Two authors independently screened titles, abstracts, keywords, and full texts to ensure relevance, excluding studies on non-lung organoids, non-organoid methods, and unrelated fields like environmental science, ecology, agriculture, and water resources.

### 2.2 Bibliometric analysis

**Figure 1:**
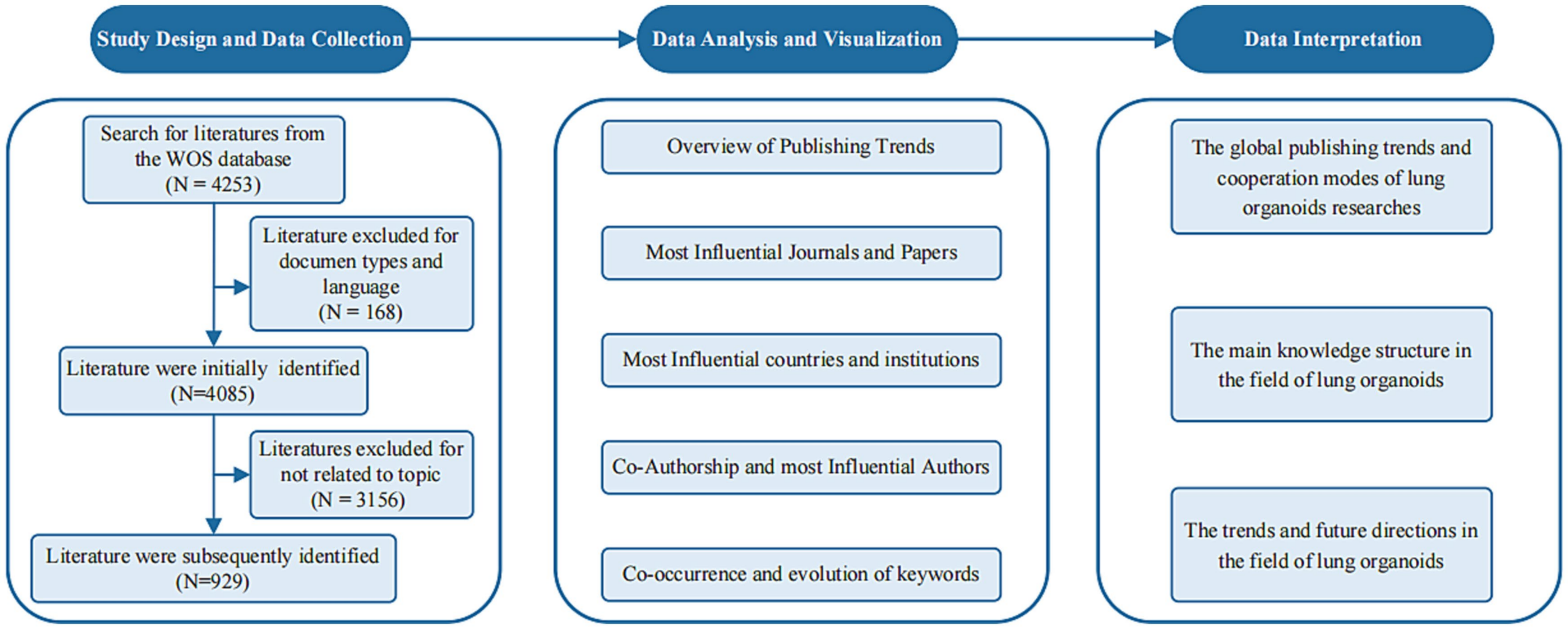
Flow chart of bibliometric analysis.

Figure 1 outlines the bibliometric analysis process. To address the Introduction’s questions, analysis focused on annual scientific output, sources, countries, institutions, authors, and keywords. Bradford’s and Price’s laws identified core journals and authors (12). Specifically, Bradford’s law posits that the core journals within a given field account for one-third of the total publications, whereas Price’s law asserts that authors with three or more publications are classified as core contributors. In the data collection phase, 4,253 articles were retrieved, with 929 valid ones selected after screening. Data was exported from WoSCC and cleaned. In our dataset, some articles lacked year information, which we retrieved from the database. Articles missing author keywords were supplemented with “keyword plus” for better information extraction. We performed necessary data splitting and merging. In keyword co-occurrence analysis, we standardized synonyms like “coronavirus disease 19” to “COVID-19” and “tumor” to “cancer,” removing irrelevant terms like “entry.” For country analysis, we unified different spellings (e.g., “peoples r china,” “taiwan,” “china”). In author analysis, we clarified authors with similar names by using full names, changing “Li, Y” to “Li, Yu” or “Li, Yue.”

The processed data were analyzed using R (version 4.2.1) and VOSviewer (version 1.6.18). R supported both qualitative and quantitative analysis of research trends, employing line charts to mark significant time points. The Bibliometrix package allowed for in-depth examination of publication characteristics, including citation counts, sources, and author identities, as well as evaluating author impact using the H-index and assessing journal quality with the 2023 impact factor (IF). R also produced dumbbell charts to monitor research hotspots and forecast trends. VOSviewer was employed to perform a co-authorship analysis, which illuminated collaboration patterns among countries and authors, alongside a keyword co-occurrence analysis aimed at identifying emerging research fronts. The findings were visualized utilizing the bibliometric mapping software SCImago Graphica. In these visual representations, the size of each node corresponds to the magnitude of the parameter (e.g., publication counts for countries or authors, frequency of keyword occurrences), while the connecting lines illustrate relationships, primarily quantified through total link strength (TLS). The thickness of the lines or the intensity of the red coloration of the nodes increase proportionally with the number of interconnections. Distinct colors are employed to differentiate various clusters for both nodes and lines.

## 3 Results

### 3.1 An overview of publishing trends

**Figure 2:**
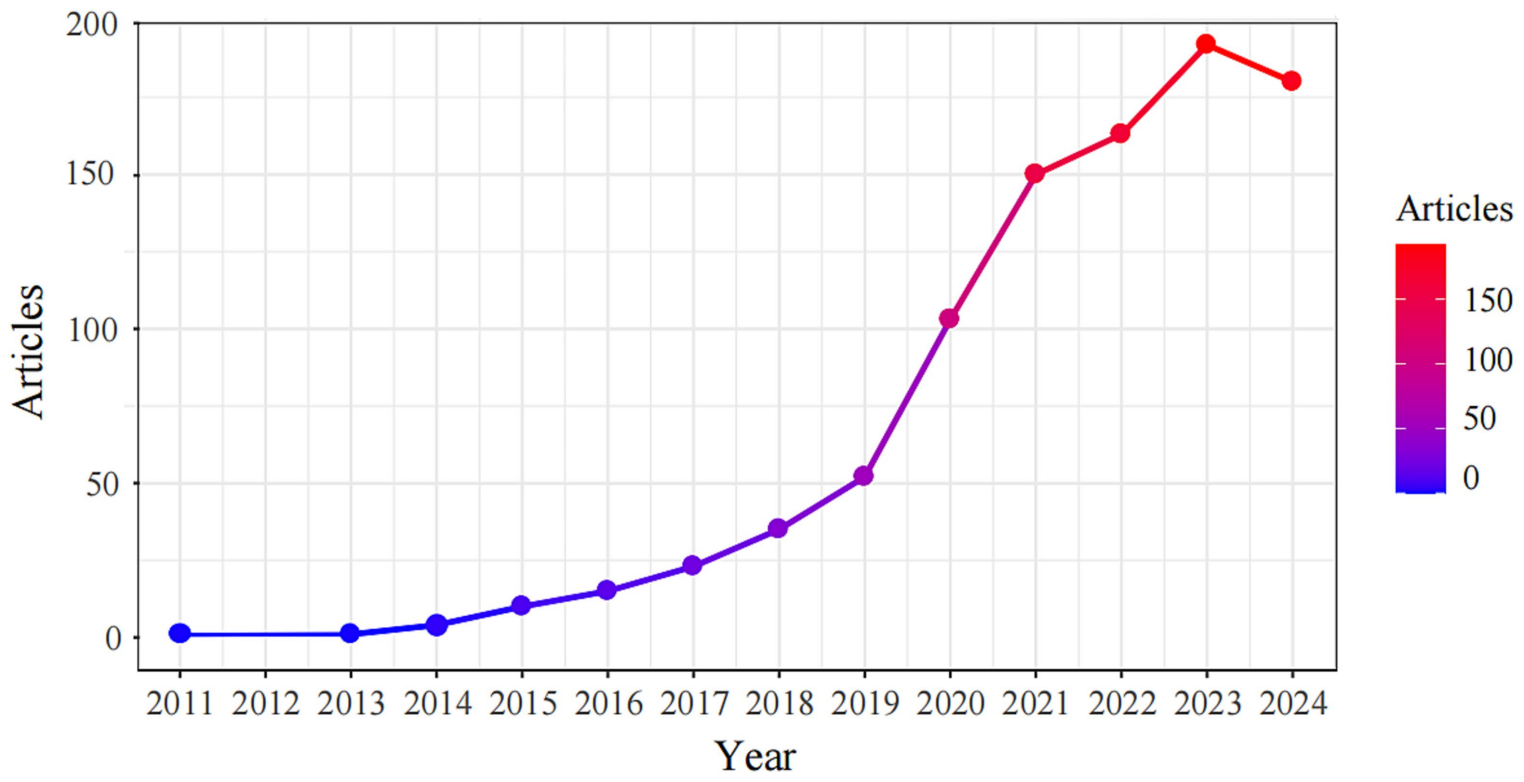
Distribution of publications from 2011 to 2024.

A total of 929 papers on lung organoids, comprising 697 articles (75.03%) and 232 reviews (24.97%), were identified, with 45,522 references. Figure 2 illustrates the publication trend, showing a gradual increase from one paper in 2011 and 2013 to a rapid rise post-2019, reaching 192 papers in 2023 and 180 in 2024, reflecting a 49.1% annual growth rate. By December 31, 2024, the total number of papers had reached 929.

### 3.2 Top 10 influential journals and papers

Table 2 highlights the top 10 influential journals by citation count. Nature leads with 2,675 citations from eight publications, followed by Cell (2,058 citations) and Cell Stem Cell (1,634 citations). Journals marked with an asterisk, including Nature, Cell Stem Cell, and others, are key sources for lung organoid research per Bradford’s law. Nature Communications, Scientific Reports, and Plos one are open-access (OA) journals. Table 3 shows the top 10 lung organoid articles, with citations ranging from 257 to 1,265. The most cited is “Modeling Development and Disease with Organoids” from Cell (2016) with 1,265 citations, followed by “SARS-CoV-2 pathogenesis” with 474 citations.

**Table 2:** Top 10 journals ranked by the number of citations in the lung organoids research field.

Rank	Source	TC	TA	IF	Category
1	Nature*	2,675	8	50.5	Multidisciplinary
2	Cell	2,058	4	45.5	Biochemistry
3	Cell Stem Cell *	1,634	8	19.8	Biochemistry
4	P NATL ACAD SCI USA*	1,583	9	9.4	Multidisciplinary
5	Nature Communications*△	1,392	24	14.7	Multidisciplinary
6	Science	1,321	1	44.7	Multidisciplinary
7	Scientific Reports*△	1,079	10	3.8	Multidisciplinary
8	AM J PHYSIOL-LUNG C*	969	21	3.6	Medicine
9	Plos one*△	942	10	2.9	Multidisciplinary
10	Development	916	7	3.7	Biochemistry

X *, the journal is the core resource (classified by Bradford Law) of lung organoids research; X△, the journal is open access; TC, the total number of citations; TA, the total number of articles; IF, the impact factor in 2023; P NATL ACAD SCI USA, Proceedings of the National Academy of Sciences of the United States of America; AMJ PHYSIOL-LUNG C, American Journal of Physiology-Lung Cellular and Molecular Physiology.

**Table 3:** Top 10 articles according to the number of citations.

Rank	Title	Journal	Citations	Year
1	Modeling Development and Disease with Organoids (31)	Cell	1,265	2016
2	SARS-CoV-2 pathogenesis (118)	Nature Reviews Microbiology	474	2022
3	In vitro generation of human pluripotent stem cell-derived lung organoids (17)	eLife	422	2015
4	Generation of Tumor-Reactive T Cells by Co-culture of Peripheral Blood Lymphocytes and Tumor Organoids (49)	Cell	389	2018
5	Long-term expanding human airway organoids for disease modeling (20)	EMBO Journal	332	2019
6	Regeneration of the lung alveolus by an evolutionarily conserved epithelial progenitor (119)	Nature	309	2018
7	A three-dimensional model of human lung development and disease from pluripotent stem cells (26)	Nature Cell Biology	279	2017
8	Integrin alpha 6 beta 4 identifies an adult distal lung epithelial population with regenerative potential in mice (120)	Journal of Clinical Investigation	276	2011
9	Organoid-on-a-chip and body-on-a-chip systems for drug screening and disease modeling (121)	Drug Discovery	262	2016
10	Multi-tissue interactions in an integrated three-tissue organ-on-a-chip platform (122)	Scientific Reports	257	2017

### 3.3 Top 10 influential countries and institutions

**Figure 3:**
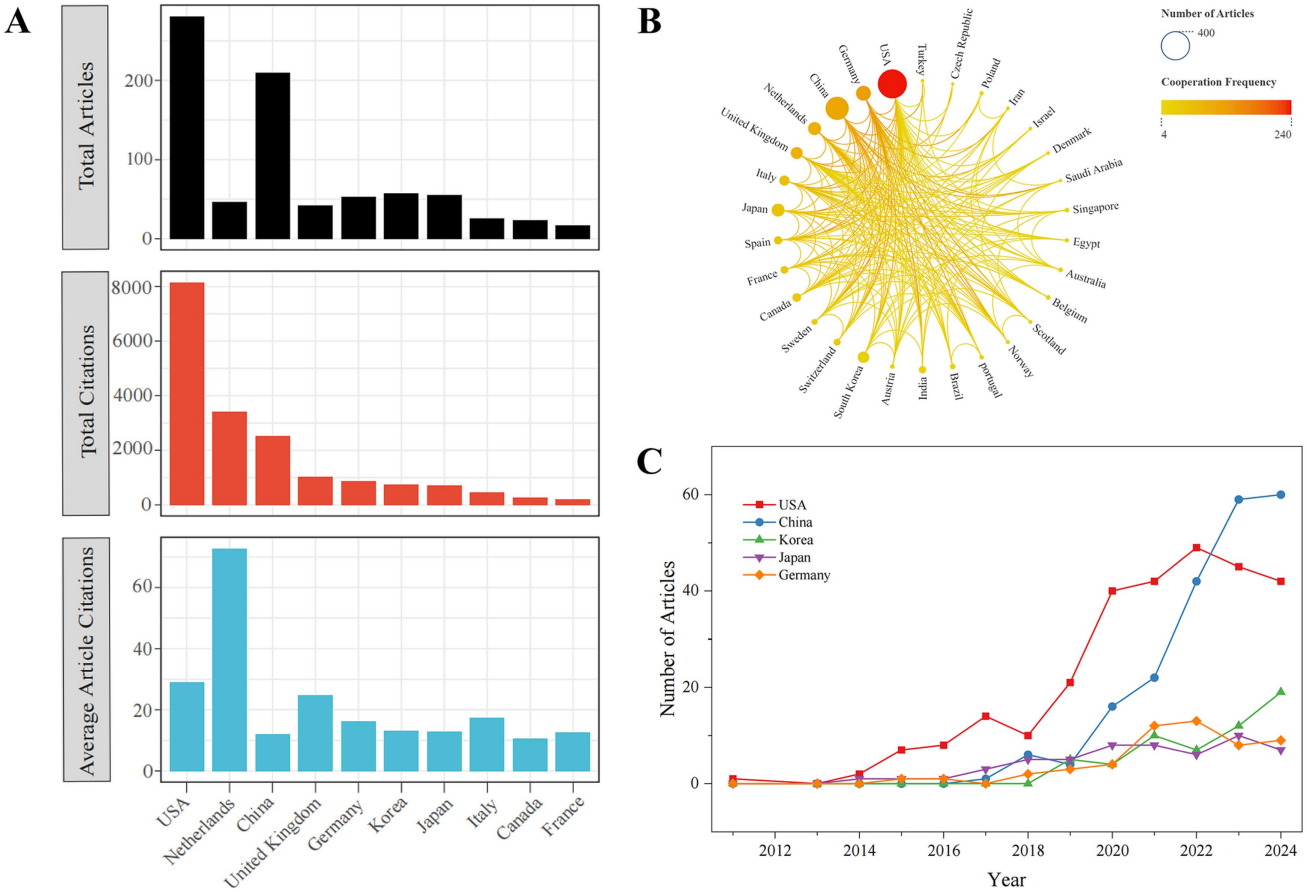
The analysis of countries related to lung organoids. **(A)** Total articles, total and average number of citations in the top 10 most highly cited countries. **(B)** Research collaboration between countries. Nodes indicate the number of national publications, with larger nodes signifying higher output. A redder node suggests more international collaboration. Lines represent cooperative relationships between countries. **(C)** Top 5 countries according to annual scientific productions.

The study analyzed lung organoid research in 55 countries. We identified the top 10 countries by total citations and calculated both the total articles and average citations for each (Figure 3A). The USA led with 8,155 citations, followed by the Netherlands (3,421) and China (2,529). The Netherlands had the highest average citations per article (72.80), with the USA second (29.00). China produced more papers and citations than most, except the USA and Netherlands, but had a lower average citation rate. As Figure 3B shows, the USA leads in collaboration, partnering with 29 countries and achieving the highest TLS (240), followed by Germany, China, and the Netherlands. Top research producers include the USA (281), China (210), Korea (57), Japan (55), and Germany (53). Since 2014, the USA’s annual publications have risen significantly, with China’s increase beginning in 2017, while the other three countries have shown steady growth (Figure 3C).

Worldwide,1,638 institutions are engaged in lung organoid research. The influence of these institutions was evaluated based on article citations. The top 10 most influential institutions, primarily from the USA and the Netherlands, have the highest citations. The University Medical Center Utrecht in the Netherlands leads with 2,083 citations, followed by the Royal Netherlands Academy of Arts and Sciences (1,597), University of California, San Francisco (1,431), and Harvard Medical School (1,381). The remaining four institutions have fewer than 1,000 citations (Table 4).

**Table 4:** Top 10 institutions according to the total number of citations in the lung organoids research field.

Rank	Institution	Country	TC	TA	TC/TA
1	University Medical Center Utrecht	Netherlands	2,083	11	189.36
2	Royal Netherlands Academy of Arts and Sciences	Netherlands	1,597	9	177.44
3	University of California, San Francisco	USA	1,431	17	84.18
4	Harvard Medical School	USA	1,381	29	47.62
5	University of Michigan	USA	1,288	25	51.52
6	University of Pennsylvania	USA	1,207	21	57.48
7	Princess Maxima Center for Pediatric Oncology	Netherlands	939	5	187.8
8	Duke University	USA	933	14	66.64
9	University of Cambridge	UK	887	21	42.24
10	Wake Forest School of Medicine	USA	772	8	96.5

TC, the total number of citations; TA, the total number of articles.

### 3.4 Co-authorship and top 10 influential authors

**Figure 4:**
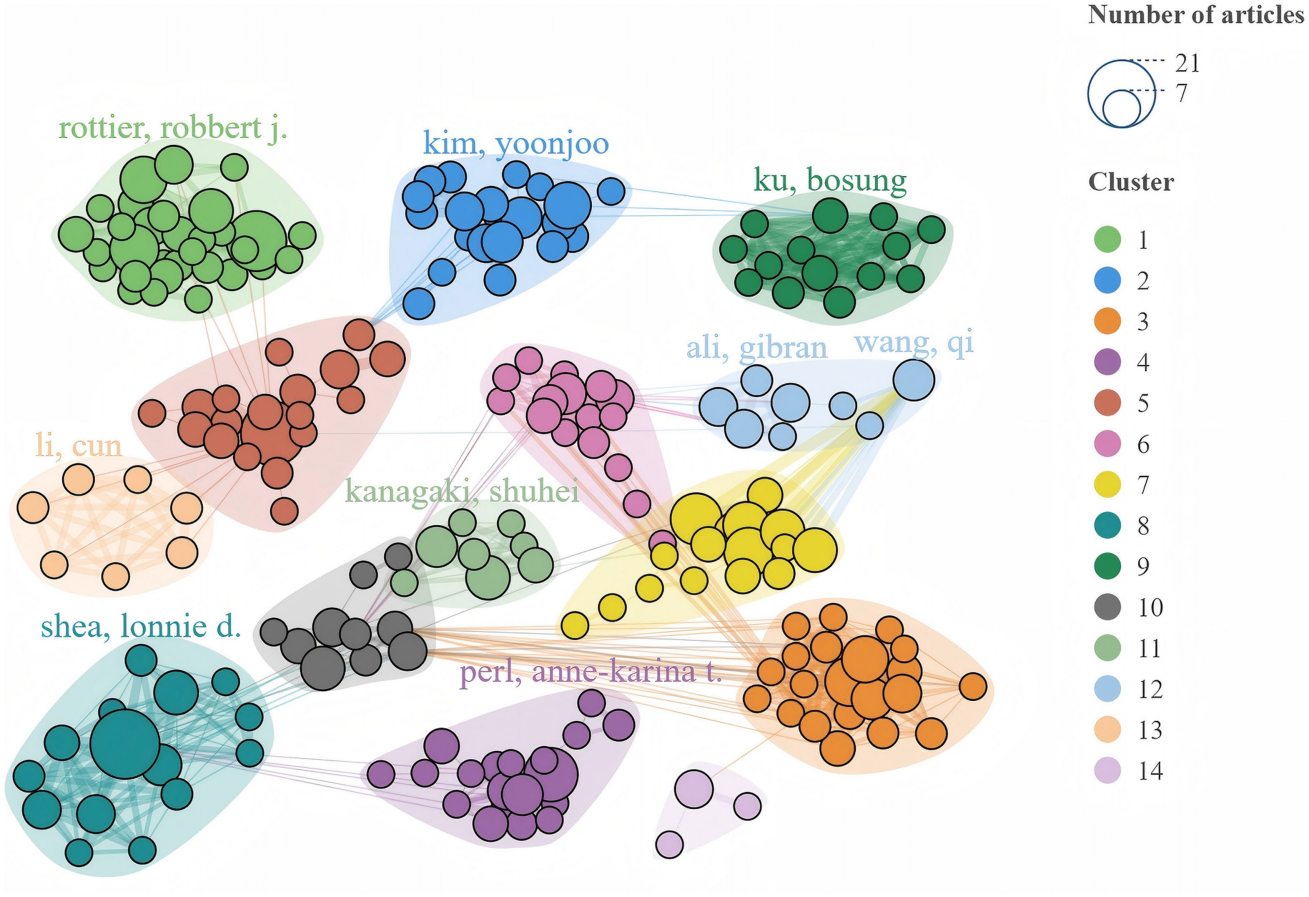
Network of core authors’ cooperation in the lung organoids research field.

The 929 papers involved 7,263 authors, with 13 being single-author works. The H-index (13–15), a measure of research impact, highlighted Clevers H as the most influential author, with the highest citations in the WoSCC database. The top 10 authors by H-index included five from the USA, two from the Netherlands, two from the UK, and one from Korea. There are 303 core authors, defined by Price’s law as those with at least three publications. The top three authors by TLS were Spence JR (71), Chen HY (65), and Bellusci S (64) (Table 5). Figure 4 reveals that the co-authorship network of 208 authors is split into 14 clusters, highlighting a fragmented and loosely concentrated collaboration, suggesting that the lung organoid field lacks a unified collaboration model.

**Table 5:** Top 10 influential authors in the lung organoids research field.

Rank	Author	H-index	TC	TA	PY-start	Country
1	Clevers H	13	2,711	21	2016	Netherlands
2	Spence JR	12	1,158	20	2015	USA
3	Lee JH	9	700	13	2016	UK
4	Morrisey EE	9	698	13	2016	USA
5	Choi J	8	388	12	2016	UK
6	Dye BR	8	838	8	2015	USA
7	Gosens R	8	190	16	2018	Netherlands
8	Hogan BLM	8	994	8	2014	USA
9	Kim JH	8	257	12	2020	Korea
10	Wang Y	8	405	14	2014	USA

TC, the total number of citations; TA, the total number of articles; PY_start, first year published.

### 3.5 The co-occurrence and evolution of keywords

**Figure 5:**
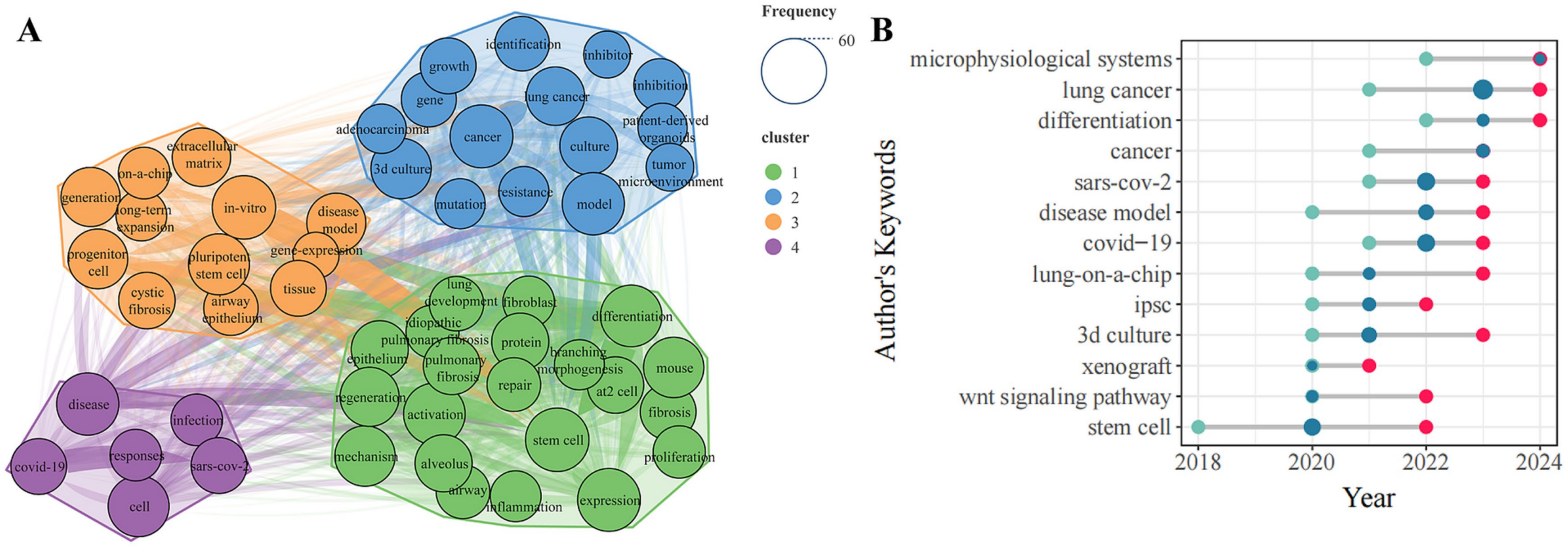
The analysis of keywords related to lung organoids. **(A)** Co-occurrence of high-frequency keywords. Node labels show keywords, with sizes representing their frequency. Different colored nodes indicate clusters, and line thickness shows connection strength. **(B)** Temporal trends in high-frequency keywords. The *X*-axis shows the year, while the *Y*-axis represents author keywords over time. Green, red, and blue points denote the first quantile, third quantile, and median publication years for each keyword, respectively.

A total of 3,745 keywords were detected in 929 articles published on lung organoid research. VOSviewer set a minimum occurrence threshold of 25 for keywords, leading to 57 keywords being eligible for network visualization analysis. The top 10 were organoids (229), stem cell (188), differentiation (155), expression (132), *in vitro* (131), cancer (125), lung (125), lung cancer (124), disease (99), and pluripotent stem cell (99). Figure 5A shows that 54 keywords, excluding subject words, were grouped into four clusters based on disease types: Cluster 1 (green) for idiopathic pulmonary fibrosis (IPF), Cluster 2 (blue) for lung cancer, Cluster 3 (orange) for cystic fibrosis (CF), and Cluster 4 (purple) for COVID-19.

Figure 5B shows the temporal trends in high-frequency keywords. The top five keywords are “lung cancer,” “SARS-CoV-2,” “COVID-19,” “stem cell,” and “3D-culture.” A rightward red dot indicates a later emergence of research trends. “Stem cell” is the earliest and most consistently used keyword, while “microphysiological systems” and “differentiation” are the newest. Recently, lung organoid research has focused on “microphysiological systems,” “lung cancer,” “differentiation,” “SARS-CoV-2,” “disease model,” “COVID-19,” “lung-on-a-chip,” and “3D culture.”

## 4 Discussion

### 4.1 Answer for Q1: what are the global publishing trend and cooperation modes of lung organoids?

In 2014, the first airway organoids were created from induced pluripotent stem cells (iPSCs), initiating lung organoid research (16). Since then, publications have nearly doubled each year, leading to significant advancements such as human-derived lung organoids and those replicating completed airway and alveolar structures (17, 18). By 2019, research shifted from structural modeling to applications in lung disease modeling, drug screening, and therapeutic studies (19–21). The COVID-19 pandemic in 2020 further propelled this field, making lung organoids crucial for studying severe acute respiratory syndrome coronavirus-2 (SARS-CoV-2) entry, replication, inflammatory responses, and antiviral drug testing (e.g., remdesivir) (22–25), which fostering interdisciplinary collaboration in personalized medicine.

The USA and the Netherlands lead in lung organoid research, thanks to strong research foundations and resources. Key U. S. institutions like University of California, San Francisco, Duke University, and Harvard Medical School drive significant progress in lung development, disease modeling, regenerative medicine, and viral infection studies (26–28). The USA also fosters interdisciplinary collaboration across biomedical science, engineering, and informatics, enhancing clinical translation of lung organoids with technologies like microfluidics, 3D printing, and gene programming (29, 30). Despite producing fewer academic publications than the United States, the Netherlands boasts most representative and influential researchers and institutions. Notably, Hans Clevers and his team pioneered the use of stem cell technology to create functional lung organoids, offering a new platform for studying and treating lung diseases like cancer and fibrosis (31). The University Medical Center Utrecht, with which he is affiliated, is acknowledged as a preeminent institution in the field of global lung organoid research. Beyond its role in advancing diverse lung disease organoid models, the institution is dedicated to employing these models to investigate respiratory viral infections, including COVID-19, as well as lung immune response (32, 33). Research efforts have also emerged from China, and there has been a rise in participation from new researchers and institutions.

International collaboration in lung organoid research is limited, with prominent authors and institutions focusing mainly on domestic partnerships. This is due to the competitive nature and academic challenges of the field, which encourage teams to keep their findings private. Additionally, differing ethical and regulatory standards across countries hinder cross-national cooperation. Moreover, the development of lung organoids involves complex technologies and experimental conditions, with varying research progress among teams affecting collaboration. Lung organoid research is prominent, with significant findings published in top journals like Nature, Cell, and Cell Stem Cell, but most of these are not OA, limiting wider dissemination. Due to limited access to lung organoid research, we urge the publication of studies in OA formats to improve their reach. This approach boosts transparency, reproducibility, and impact, allowing a wider global research community to access and validate the findings. The OA formats also promote interdisciplinary collaboration across fields like medicine, biology, pharmacology, and materials science, advancing the clinical use of lung organoids.

### 4.2 Answer for Q2: what is the main knowledge structure in the field of lung organoids?

The main knowledge structure was identified based on co-occurrence analysis of high-frequency keywords. Between 2011 and 2024, lung organoid research is divided into four clusters related to lung diseases, as shown in Figure 5A.

#### 4.2.1 Cluster 1 (green): IPF

The IPF is a fatal lung disease characterized by progressive scarring of lung tissue that impairs breathing and is irreversible, with a poor prognosis (34). Advances in organoid technology enable the creation of 3D lung tissue models *in vitro*, which are more effective than bleomycin-induced animal models for studying IPF’s complex cell interactions and are better for preclinical research (35). Abnormal regeneration of alveolar epithelial cells, particularly alveolar type 2 (AT2) cells, is crucial in IPF development. Studies show that disrupted epithelial-stromal interactions impair regeneration, leading to alveolar damage (36). As IPF progresses, AT2 cells lose their regenerative capacity due to functional issues and differentiation changes (37). Risk factors for IPF include aging and mucin 5B (MUC5B) promoter variants (38). In AT2 cells, adenine nucleotide translocases 1 (ANT1) staining increases senescence markers (39), and co-culturing with fibrotic fibroblasts induces cystic growth and MUC5B expression (40). Interleukin-11 (IL-11) is also identified as a potential therapeutic target, as it impedes alveolar epithelial regeneration (41).

Lung organoids are valuable for drug screening in IPF. Gokey et al. (42) discovered that retinoic acid aids alveolar cell repair and fibroblast regeneration, suggesting a potential treatment. Ptasinski et al. (43) found that nintedanib and pirfenidone reduce fibrosis markers but do not fully reverse epithelial changes, highlighting the need for therapies targeting the alveolar epithelium. Moreover, integrating clustered regularly interspaced short palindromic repeats/CRISPR-associated protein 9 (CRISPR/Cas9) with lung organoids could become a new trend, which allows scientists to simulate IPF-related gene mutations, potentially advancing precise gene therapy strategies (21, 44).

#### 4.2.2 Cluster 2 (blue): lung cancer

Lung cancer ranks among the foremost causes of cancer-related mortality, with its substantial heterogeneity, tumor cell plasticity, and the dynamic regulation of the tumor microenvironment (TME) posing significant challenges to the formulation of effective treatment strategies (45). Patient-derived organoids (PDOs) have overcome the limitations inherent in traditional cell line models by preserving tumor heterogeneity, thereby providing an optimal platform for elucidating the mechanisms underlying lung cancer development and evaluating preclinical treatment responses (46, 47). PDOs that incorporate the TME can simulate intercellular signaling and immune surveillance mechanisms through a reconstruction method (48, 49), or retaining critical components of the *in vivo* microenvironment, such as the original tumor’s extracellular matrix and vascular mimetics, facilitating the examination of responses to immunotherapy and targeted therapies through a whole method approach (50, 51).

Lung cancer organoids (LCOs) are vital for developing new drug targets and conducting large-scale screenings. Zhang et al. (52) found significant differences in drug sensitivity through cell viability tests on lung cancer assembloids and organoids. Neal et al. (51) used an air-liquid interface PDO model from patient tumor fragments to evaluate PDOs’ response to nivolumab, highlighting its potential for immunotherapy screening. Drug resistance poses a significant challenge in cancer treatment. Luan et al. (53) demonstrated that fibroblast-secreted factors enhance tumor resistance to adagrasib using an LCO-cancer-associated fibroblasts co-culture model. Wang et al. (54) showed that inflammatory factors, particularly SAA secreted by cancer stem cells, contribute to cisplatin resistance in lung cancer models. Beyond co-culture systems, CRISPR-Cas9 technology is pivotal in uncovering resistance mechanisms in LCOs. Pfeifer et al. (55) used genome-wide CRISPR screening in LCOs to confirm the Hippo signaling pathway as a potential target to prevent osimertinib resistance.

#### 4.2.3 Cluster 3 (orange): CF

The CF is a genetic disorder resulting from mutations in the cystic fibrosis transmembrane conductance regulator (CFTR) gene, and is marked by airway mucus obstruction, chronic inflammation, and pulmonary fibrosis (56). While traditional functional imaging of intestinal organoids (FIS) is frequently employed to evaluate the specific effects of CFTR modulators on rare mutations (57, 58), research indicates that lung organoids offer superior phenotypic advantages in replicating CF pathological characteristics, including thickened mucus layers and electrolyte transport deficiencies (20, 59, 60). Later studies confirmed that lung organoids derived from human iPSCs are effective for assessing CFTR channel functionality using FIS assays, with some findings suggesting they may react more strongly to amiloride compared to airway organoids (61). Gene editing technologies, particularly CRISPR/Cas9, are essential for modeling lung organoids in CF, as they can effectively correct the CFTR gene and restore chloride ion secretion in patient-derived organoids, paving the way for personalized gene therapy (62–64). The incorporation of lung organ-on-a-chip technology enhances the model’s ability to replicate physiological conditions, such as interactions between CF epithelial cells and neutrophils, mucus accumulation, and ciliary dysfunction (65). CF lung organoid research is still in its early stages, mainly concentrating on genetics. Future research could incorporate tissue engineering to explore CFTR defects’ impact on immune responses and lung inflammation (66).

#### 4.2.4 Cluster 4 (purple): COVID-19

COVID-19, caused by SARS-CoV-2, is highly transmissible with a long incubation period and affects multiple organs (67, 68). Lung organoids, as 3D models, mimic human viral infections and help study virus-host interactions (24, 69). They are used to examine changes in respiratory cells under different viral loads, with angiotensin-converting enzyme 2 (ACE2) and transmembrane protease serine 2 (TMPRSS2) expression levels influencing susceptibility. Katsura et al. (70) discovered that ACE2 expression in AT2 cells of lung stem cell-derived alveolar spheroids facilitates SARS-CoV-2 infection. Mykytyn et al. (71) demonstrated that the virus’s multibasic cleavage site (MBCS) boosts serine protease-mediated entry, such as TMPRSS2, into airway organoids. Furthermore, Lung organoids capture host genetic diversity, highlighting variations in viral responses across different ages, sexes, and races, which supports personalized treatment strategies (72).

Lung organoid research closely replicates the human physiological environment, reducing interspecies variability and aiding rapid drug and vaccine development for diseases like COVID-19. Current studies focus on antiviral drugs targeting viral entry and replication using lung organoids from human pluripotent stem cells. High-throughput screening has identified SARS-CoV-2 entry inhibitors such as imatinib, mycophenolic acid and quinacrine dihydrochloride (73). Wang et al. (74) found that the interferon-stimulated gene cholesterol-25-hydroxylase (CH25H) blocks SARS-CoV-2 entry in human lung organoids. Additionally, androgen-blocking drugs show potential by lowering ACE2 expression and protecting lung organoids from infection (75). Regarding viral replication, low-dose interferon lambda 1, remdesivir, and human neutralizing antibodies effectively inhibit SARS-CoV-2 replication in lung organoids (33, 76), but combination therapies may yield better results (77, 78). Studies show that remdesivir combined with nelfinavir or camostat mesylate has a synergistic effect against SARS-CoV-2. As more lung organoid research data emerges, integrating artificial intelligence (AI) and big data analysis will help identify drug targets, immune biomarkers, and predict long-term COVID-19 effects, becoming a mainstream research trend (79).

### 4.3 Answer for Q3: what are the future prospectives and challenges in the field of lung organoids?

#### 4.3.1 Future prospectives in the field of lung organoids

From an overall development perspective, early research on lung organoids primarily focused on model construction. The Wnt signaling pathway is crucial for lung development, balancing stem cell renewal and differentiation into mature lung cells like alveolar epithelial cells in lung organoids (80, 81). Recent research on lung organoids has increasingly focused on disease modeling, particularly lung cancer and COVID-19. For lung cancer, the emphasis is on using PDOs with the tumor microenvironment. In particular, co-culturing immune cells with PDOs allows for modeling tumor-immune interactions, which is valuable for cancer immunotherapy and developing new treatments. However, most studies focus on exogenous immune components, particularly T cell activation (82, 104). Moreover, it is crucial to validate the correlation between PDO responses and patient clinical outcomes to establish these co-culture platforms as clinically relevant preclinical models for immunotherapy. Therefore, more comprehensive investigations into these areas are necessary in future research endeavors. Precision treatments require large-scale clinical trials for validation. The pandemic prompted scientists to focus on SARS-CoV-2 infection mechanisms, lung injury, immune responses, and inflammation, leading to the swift creation of virus-specific models for personalized drug screening (83–86). Although the acute phase of the pandemic has subsided, issues such as SARS-CoV-2 variant infections (87–89), and co-infections with other viruses (90) remain subjects of future significant attention.

In addition to disease modeling, research also focuses on microphysiological systems, or organ-on-a-chip, which utilize microfluidic flow to cultivate cells in organ-like structures. This allows for precise control of biochemical and biophysical conditions, replicating specific cellular behaviors and ensuring consistent lung microenvironment reproduction (91). These systems improve lung organoid stability and address issues such as expansion challenges and incomplete tumor microenvironment characterization, making them valuable in lung organoid drug treatment research (92, 93). Zhang et al. (94) designed a microfluidic chip with an adenosine triphosphate (ATP) sensor for real-time lung organoid monitoring, while Wu et al. (95) created an automated system for single-cell sequencing of lung cancer organoids to identify mutations. Although promising, microphysiological systems remain in early development, needing future standardized evaluations and interdisciplinary collaboration.

#### 4.3.2 Future challenges in the field of lung organoids

Lung organoid research holds significant potential for biomedical and clinical applications but faces challenges such as standardization, disease modeling accuracy, and ethical and safety issues.

The primary challenge is standardization in lung organoid research, which faces issues with culture conditions, phenotype evaluation, biobank construction, and data analysis. Variations in these areas lead to inconsistencies in morphology, functionality, and disease modeling (96). Additionally, diverse data analysis methods cause inconsistent results, limiting reproducibility and clinical relevance. To address these issues, a unified consensus within the lung organoid field is needed. Besides, creating a standardized biobank for biological samples, integrating data from various sources, and facilitating data sharing is crucial. One study has identified standardization issues in organoid production and suggested improvements in cell lines and culture media (97). Zhang et al. (98) developed a new platform using Pluronic F-127, enhancing batch consistency and reducing costs. However, significant progress is still needed to fully address the standardization challenges.

The second challenge is achieving accurate disease modeling with lung organoids. This difficulty stems from their immature development, which hinders the replication of the lung’s complex architecture and physiological processes, and from the diverse nature of diseases, making it hard to mimic individual patient differences. To overcome these issues, optimizing culture protocols is essential, utilizing tissue engineering techniques like biomaterial scaffolds to better simulate lung biomechanics and specific disease states (29, 60, 99). Furthermore, establishing a biobank with diverse live biological samples is crucial for enhancing model accuracy and capturing individual disease variations. For example, Liu et al. (50) developed patient-derived LCOs with a single-cell RNA sequencing platform to study immune responses in lung cancer. However, research mainly focuses on anti-cancer therapies. There is a need for multi-modal modeling systems that integrate existing lung organoid models and data to simulate complex disease mechanisms, facilitate targeted drug screening, and improve predictive accuracy for early disease detection and progression tracking (100).

The main challenge involves ethical and safety concerns, such as the source of cells for lung organoids, ethical issues in embryonic research, uncertainties with gene editing, potential mutations or immune reactions during organoid cultivation, and tumor risks with long-term culture and transplantation (101). Additionally, constructing a biobank or modeling systems requires informed consent, data protection, ethical considerations in animal and human research, cross-cultural ethics, genetic editing risks, and biosafety. Researchers call for strict ethical reviews, better regulation of gene editing and cell sourcing, and genomic and phenotypic monitoring to ensure organoid safety and functionality (102, 103).

### 4.4 Innovations and limitations

This study is the first to comprehensively map lung organoid research using bibliometric methods, addressing knowledge gaps and guiding future research without subjective bias. Unlike previous reviews, we identified themes through data analysis and highlighted key topics for researchers. We also detailed challenges in lung organoid research. While our analysis is comprehensive and objective, it is limited by the use of a single database, which may omit some relevant literature. However, this ensures consistency and transparency. We chose WoSCC for its extensive, high-quality academic information and rigorous journal selection criteria, ensuring data reliability and authority.

## 5 Conclusion

This study firstly reveals publication trends, global collaborations, and research hotspots of lung organoids over the past 14 years. The field of lung organoids is growing rapidly and is likely to expand further in the future. The Netherlands and the USA are undoubtedly the main drivers of global research. The research hotspots for lung organoids are disease modeling (lung cancer and COVID-19) and microphysiological systems. Standardization, the accuracy of disease modeling, and ethics and safety remain pressing challenges that need to be addressed in this field. These findings can help the research community identify emerging topics and frontiers in lung organoids and provide references for future research.

## Data Availability

The original contributions presented in the study are included in the article/supplementary material, further inquiries can be directed to the corresponding author.
